# Cost saving analysis of specialized, eHealth-based management of patients receiving oral anticoagulation therapy: Results from the thrombEVAL study

**DOI:** 10.1038/s41598-021-82076-9

**Published:** 2021-01-28

**Authors:** Lisa Eggebrecht, Paul Ludolph, Sebastian Göbel, Marina Panova-Noeva, Natalie Arnold, Markus Nagler, Christoph Bickel, Michael Lauterbach, Roland Hardt, Hugo ten Cate, Karl J. Lackner, Christine Espinola-Klein, Thomas Münzel, Jürgen H. Prochaska, Philipp S. Wild

**Affiliations:** 1 Preventive Cardiology and Preventive Medicine, Center for Cardiology, University Medical Center of the Johannes Gutenberg University Mainz, Langenbeckstraße 1, Mainz, 55131 Germany; 2 Center for Thrombosis and Hemostasis, University Medical Center of the Johannes Gutenberg-University Mainz, Mainz, Germany; 3 Department of Psychiatry and Psychotherapy, University Medical Center of the Johannes Gutenberg University Mainz, Mainz, Germany; 4 Center for Cardiology – Cardiology I, University Medical Center of the Johannes Gutenberg-University Mainz, Mainz, Germany; 5 German Center for Cardiovascular Research (DZHK), Partner Site Rhine Main, Mainz, Germany; 6 Department of Medicine I, Federal Armed Forces Central Hospital Koblenz, Koblenz, Germany; 7 Department of Medicine 3, Barmherzige Brüder Hospital, Trier, Germany; 8 Center for General Medicine and Geriatric Medicine, University Medical Center Mainz, Johannes Gutenberg University-Mainz, Mainz, Germany; 9 CARIM/ Department of Vascular Medicine, Heart and Vascular Center, University Medical Center Maastricht, Maastricht, The Netherlands; 10 Institute of Clinical Chemistry and Laboratory Medicine, University Medical Center of the Johannes Gutenberg University Mainz, Mainz, Germany

**Keywords:** Health care, Health care economics

## Abstract

To evaluate the cost-saving of a specialized, eHealth-based management service (CS) in comparison to regular medical care (RMC) for the management of patients receiving oral anticoagulation (OAC) therapy. Costs of hospitalization were derived via diagnosis-related groups which comprise diagnoses (ICD-10) and operation and procedure classification system (OPS), which resulted in OAC-related (i.e. bleeding/ thromboembolic events) and non-OAC-related costs for both cohorts. Cost for anticoagulation management comprised INR-testing, personnel, and technical support. In total, 705 patients were managed by CS and 1490 patients received RMC. The number of hospital stays was significantly lower in the CS cohort compared to RMC (CS: 23.4/100 py; RMC: 68.7/100 py); with the most pronounced difference in OAC-related admissions (CS: 2.8/100 py; RMC: 13.3/100 py). Total costs for anticoagulation management amounted to 101 EUR/py in RMC and 311 EUR/py in CS, whereas hospitalization costs were 3261 [IQR 2857–3689] EUR/py in RMC and 683 [504–874] EUR/py in CS. This resulted in an overall cost saving 2368 EUR/py favoring the CS. The lower frequency of adverse events in anticoagulated patients managed by the telemedicine-based CS compared to RMC translated into a substantial cost-saving, despite higher costs for the specialized management of patients.

Trial registration: ClinicalTrials.gov, unique identifier NCT01809015, March 8, 2013.

## Introduction

Oral anticoagulation (OAC) therapy is effective at preventing stroke and systemic embolism in patients with atrial fibrillation or venous thrombosis, and reduces morbidity and mortality in individuals with established thromboembolic disease^1^. Vitamin K antagonists (VKA) and direct acting anticoagulants (DOAC) are currently the most commonly used anticoagulant drugs, of which both require a deliberate management of therapy (e.g. due to comorbidities such as renal failure or heart failure). Although DOAC offer similar (or better) effectiveness, safety, and convenience to VKA^2–5^, there is still a substantial amount of patients receiving VKA^6,7^.

Treatment with VKA merits special attention: Interactions of VKA with co-medication, food or certain pharmacogenomics variations (e.g. *VKORC1, CYP2C9, CYP4F2*) can lead to fluctuations of the international normalised ratio (INR)^8^, and subsequently increase the risk of bleeding and other clinically relevant complications^9,10^. Hence, effective and safe management of VKA treatment requires a regularly and intensive observation of patients by their physician. However, inadequate treatment of many patients implies the need for optimizing the management of oral anticoagulation^11^, and formally structured anticoagulation services providing specialized care have been shown to improve the clinical outcome of patients when compared to usual medical care^12,13^.

So far, a limited number of studies have evaluated the economic impact of an anticoagulation service. Besides the low generalizability of results due to difference in social aspects and healthcare systems from country to country, most studies lack accurate data assessment of operational cost and medical claims^14–16^. All studies stated a reduced health care expenditure of multi-disciplinary anticoagulation services compared with usual care^14–19^. However, no data is available on the cost-saving of a specialized, eHealth-based management system.

Especially against the background of the availability of (still) cost-intensive alternatives to VKA, it is of great interest to evaluate cost-efficiency of a specialized, eHealth-based management system compared with usual medical care investigating real world outcome in patients receiving VKA, which is supportive for making public health decisions.

## Results

### Baseline characteristics of study sample

Patient characteristics of both cohorts are displayed in Table 1. In total, 705 patients were managed by CS (median age: 73.0 years [IQR 63.0/80.0]; 52% male) and 1490 patients received RMC (median age: 73.0 years [IQR 65.0/79.0]; 64% male) with 465 and 1185 patient-years (py), respectively. Individuals in RMC exhibited a worse clinical profile than patients managed by CS. Overall, reasons for hospital admission did not relevantly differ between groups (Table 2): most frequent reasons for hospital admission were atrial fibrillation/flutter (12% for both cohorts) and heart failure (11.8% for RMC; 10.1% for CS).

**Table 1:** Baseline characteristics of study participants according to healthcare model.

	Regular medical care (n = 1490)	Coagulation service (n = 705)	*P* value
Age [years]	73.0 (65.0/79.0)	73.0 (63.0/80.0)	0.69
Male sex—% (n)	63.8% (951)	51.8% (365)	< 0.0001
Arterial hypertension	78.8 (1174)	75.7 (534)	0.11
Atrial fibrillation	73.6 (1088)	64.3 (453)	< 0.0001
Chronic kidney disease	23.4 (346)	15.9 (112)	< 0.0001
Chronic lung disease	22.1 (326)	16.0 (112)	0.00073
Congestive heart failure	43.0 (628)	30.2 (211)	< 0.0001
Diabetes	30.8 (459)	26.8 (187)	0.056
Dyslipidemia	55.2 (821)	43.1 (304)	< 0.0001
Family history of MI/Stroke	41.3 (615)	30.6 (216)	< 0.0001
History of bleeding	32.5 (459)	16.2 (114)	< 0.0001
History of myocardial infarct ion	22.1 (327)	11.7 (82)	< 0.0001
History of Stroke/ TIA	17.5 (260)	17.7 (125)	0.90
History of venous thromboembolism	23.3 (345)	33.4 (235)	< 0.0001
Obesity	29.7 (442)	32.5 (229)	0.20
Peripheral artery disease	23.9 (347)	11.4 (80)	< 0.0001
Smoking (current)	7.7 (115)	5.2 (37)	0.038
Tumor disease	17.8 (261)	18.9 (131)	0.51
CHA_2_DS_2_Vasc score^a^	4.13 (1.76)	3.99 (1.75)	0.087
Charlson Comorbidity Index	5.92 (2.34)	5.31 (2.39)	< 0.0001
HAS-BLED score^a^	2.87 (2.34)	2.61 (1.31)	< 0.0001

Data are expressed as the relative and absolute frequency for binary variables; all information is displayed for baseline frequency.

^a^Calculated for patients with atrial fibrillation only.

**Table 2:** Event classifications according to healthcare model.

	Regular medical care	Coagulation service	*P* value
*Main diagnosis according to International Classification of Diseases (ICD)—% (n)*			
Angina pectoris (I20)	2.6 (21)	6.4 (7)	0.057
Atherosclerosis (I70)	3.7 (30)	0.0 (0)	0.08
Atrial fibrillation and flutter (I48)	12.0 (98)	11.9 (13)	1.00
Chronic ischemic heart disease (I25)	2.9 (24)	2.8 (3)	1.00
Heart failure (I50)	11.8 (96)	10.1 (11)	0.72
Pneumonia, unspecified organism (J18)	2.5 (20)	4.6 (5)	0.33
Operation and procedure codes (OPS)—% (n)*			
Computer-assisted image data analysis with 3D-Evaluation (3-990)	9.7 (79)	10.1 (11)	0.74
Radio-controlled cardiac telemetry (8-933)	7.9 (64)	11.0 (12)	0.19
Transesophageal echocardiography (3-052)	12.0 (98)	7.3 (8)	0.38
Transfusion of whole blood, concentrated red cells and platelet concentrates (8-800)	13.4 (109)	8.3 (9)	0.36
Unenhanced CT scan of the skull (3-200)	8.3 (68)	13.8 (15)	0.039
*Diagnosis-related groups (DRG)—% (n)*			
Cardiac arrhythmia and conduction disturbances (F71B)	4.4 (36)	3.7 (4)	0.91
Heart failure and cardiogenic shock (F62B)	8.6 (70)	5.5 (6)	0.36
Invasive cardiological diagnostic , excluding myocardial infarction (F49G)	1.5 (12)	2.8 (3)	0.56
Syncope and collapse (F73Z)	1.8 (15)	1.8 (2)	1.00
Techniques for ventricular tachycardia catheter ablation, complex (F50A)	2.9 (24)	0.0 (0)	0.13
Techniques for ventricular tachycardia catheter ablation, non-complex (F50B)	2.9 (24)	5.5 (6)	0.26
Unstable angina pectoris without severe complications (F72B)	1.1 (9)	5.5 (6)	0.0026

Operation and procedure codes (OPS) are presented according to the German procedure classification; five most common ICD, OPS and DRGs of each cohort are presented. The shown percentages are based on the number of events in each cohort.

*Multiple designations are possible for one hospitalisation (i.e. there can be multiple OPS per event. The percentages for OPS are based on number of procedures per total events).

### Cost for anticoagulation management

Management costs stratified by treatment group are shown in Table 3. According to the statutory scale of fees for physicians^20^, INR measurements in the RMC were stated as 4.70 EUR per test. Taking into account the number of tests per year (N = 21.5) resulted in total management costs of 101 EUR per py in the RMC cohort. In the CS, combining the hourly wage of staff and time per patient contact resulted in medical specialists and nursing time of 4.58 EUR and 6.31 EUR per test, respectively. Costs for dispatch of dosage recommendation and technology were estimated to be 2.07 EUR. Total management cost amounted to 311 EUR per (person-years) py in the CS cohort. Overall, total management costs were by 210 EUR per py lower in RMC compared to CS treatment.

**Table 3:** Breakdown of hospitalisation and management costs stratified by treatment group.

	Regular medical care (n = 1490)	Coagulation service (n = 705)
Patient-years (py)	1184	465
*Cost for anticoagulation management*		
Total cost per test [EUR]	4.70	12.96
Specialised nurse per test [EUR]^a^	–	6.31
Medical specialist per test [EUR]^b^	–	4.58
Preparation and dispatch of dosage recommendation per test [EUR]	–	1.25
Usage of electronic patient files per test [EUR]	–	0.82
Number of tests per year	21.5	24.0
**Total cost for anticoagulation management per patient-year [EUR]**	101	311
*Cost for hospitalisation*		
OAC related		
Number of admissions per 100 py	13.3	2.8
Cost per admission [EUR]^c^	5063 (4308–5945)	5689 (2901–8933)
Cost per patient-year [EUR]^c^	644 (492–812)	126 (48–237)
Not-OAC related		
Preparation and dispatch of dosage recommendation Number of admissions per 100 py	55.4	20.6
Preparation and dispatch of dosage recommendation cost per admission [EUR]^c^	4945 (4505–5445)	3409 (2840–4163)
Preparation and dispatch of dosage recommendation cost per patient-years [EUR]^c^	2616 (2269–2998)	557 (407–725)
**Total costs for hospitalisation per patient-year [EUR]**	3261 (2857–3689)	683 (504–874)
**Total costs [EUR]**	3362	994

^a^Nursing time per test was assumed to be 12 min for the hourly wage of 31.55 EUR.

^b^Medical specialists time per consult was assumed to be 5 min for the hourly wage of 54.92 EUR.

^c^Confidence Intervals were generated via bootstrapping.

### Hospital admissions, costs and savings

Overall, the number of hospital stays was significantly lower in the CS cohort compared to RMC (CS: 23.4 admissions per 100 py; RMC: 68.7 admissions per 100 py; Table 3). Complications of anticoagulation accompanied with a hospital stay occurred 158 and 13-times leading to 2.8 and 13.3 admissions per 100 py of the CS and RMC cohort, respectively. Regarding OAC-unrelated hospitalisations a less marked relative difference of number of admissions was observed (CS: 20.6 admissions per 100 py; RMC: 55.4 admissions per 100 py).

**Figure 1:**
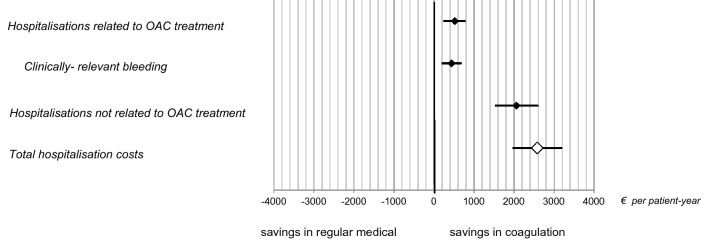
Net savings in hospitalisation costs per py for anticoagulation specific and non-specific outcomes.

Referring to OAC related hospitalisations, actual costs per hospital admission based on medical claims was slightly higher for CS, although without significant difference (CS: 5689 [IQR 2901–8933] EUR; RMC: 5063 [IQR 4308–5945] EUR; *p* = 0.70), whereas the costs for OAC unrelated hospitalisations were lower in CS (CS: 3409 [IQR 2840–4163] EUR; RMC: 4945 [IQR 4505–5445] EUR; *p* < 0.001). Net savings in hospitalisation costs per py for anticoagulation specific and non-specific outcomes are shown in Fig. 1. Greater savings in hospitalisation costs per py in CS were observed for non-OAC related events compared to OAC-related (i.e. 2059 [IQR 1544–2591] EUR vs. 518 [IQR 255–764] EUR).

Taking into consideration the rate of admissions and costs per admission, costs per py were diminished in the CS cohort for OAC-related as well as OAC-unrelated hospital admissions resulting in 5-times lower total hospitalisation costs per py in CS (CS: 683 [IQR 504–874] EUR vs. RMC: 3261 [IQR 2857–3689] EUR). In summary, total management costs per py in RMC were lower by 210 EUR compared to CS, whereas hospitalization costs per py were 2578 EUR higher. Taking into account management costs, the overall cost saving was 2368 EUR per py favouring the CS treatment.

**Figure 2:**
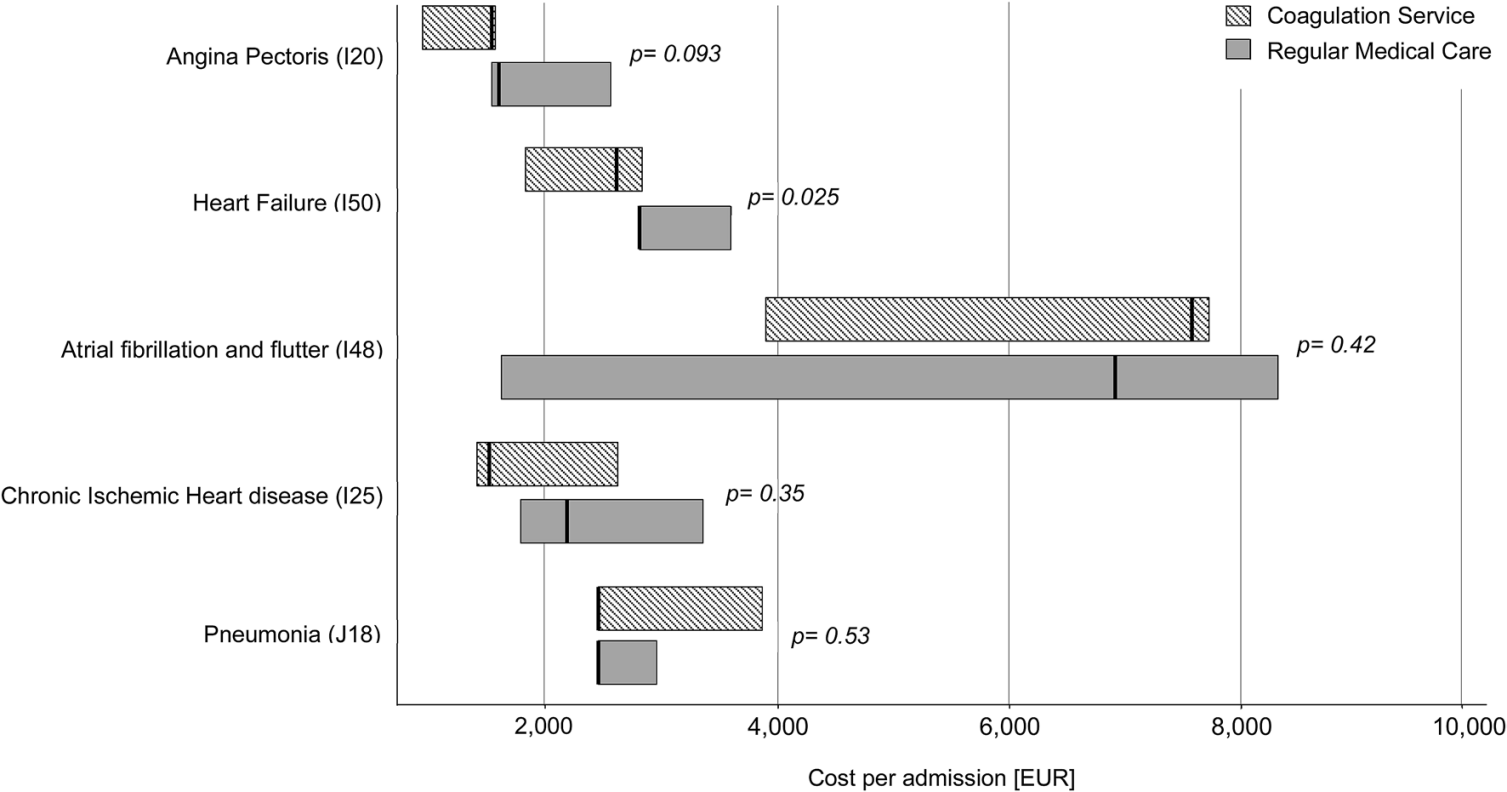
Median costs of hospitalizations for the five most common ICD-principal diagnoses stratified by healthcare model.

As displayed in Fig. 2, the main reasons for hospitalisations were diseases of the circulatory system. Hospital stays for the four most common ICD principal diagnoses (i.e. atrial fibrillation/flutter, heart failure, chronic ischemic heart disease, angina pectoris) were less costly in the CS cohort. Costs of hospitalisation stays for heart failure were significantly lower in CS (RMC: 2815 [IQR 2815–3602] EUR per admission vs. CS: 2620 [IQR 1494–2852] EUR per admission; *p* = 0.025).

### Hospitalisation costs over time

**Figure 3:**
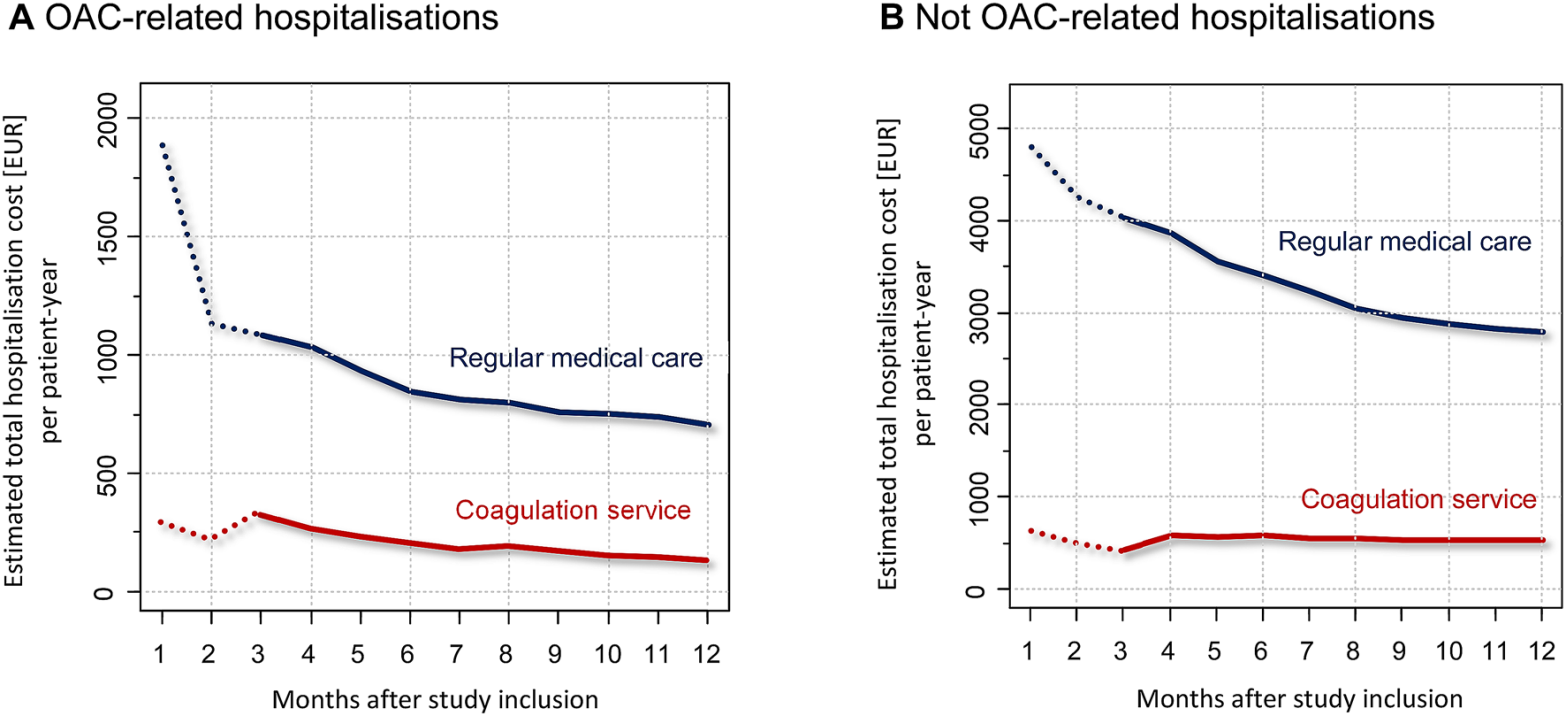
Hospitalisation costs over the study period.

Figure 3 provides an overview on average OAC-related and OAC-unrelated hospitalisation costs over the study period of 12 months. For both occasions, average hospitalization costs (comprising number of admissions and cost per admission) were considerably higher in the RMC at the start of the study. Costs over time—albeit at different levels—decreased over time in both cohorts. Referring to OAC-unrelated hospitalisations, the change of average hospitalisations costs in the CS cohort over time was comparatively small. The total non-OAC-related hospitalisation costs in the CS cohort stabilised at a constant value of approximate 500 EUR per py.

### Propensity score weighted analysis

In order to account for the potential impact of differences in the clinical profile of study participants in both cohorts on cost estimates, inverse probability of treatment weighting using the propensity score was conducted. Lower costs per py for OAC-related hospitalisation in CS (CS: 139 [IQR 31–305] EUR vs. RMC: 587 [IQR 447–741] EUR) were revealed, idem for non-OAC related hospitalisations (CS: 706 [IQR 420–1058] EUR vs. RMC: 2523 [IQR 2186–2901] EUR). Total hospitalisation costs were again lower by 2266 EUR per py. Taking into account management costs for both cohorts, net saving cost were 2056 EUR per py in the CS.

## Discussion

This is the first study to evaluate the cost-saving of an eHealth based coagulation service compared with regular medical care for the management of patients receiving oral anticoagulation therapy with VKA accounting for operational costs, therapeutic outcome and the associated costs based on actual health claims data in a real-world setting. The present study demonstrated that the savings of a CS observed with regard to clinical outcome translate into substantial money saving of over 2000 EUR per py. Importantly, the positive cost-effectiveness was due to a substantial saving in both OAC-related und -unrelated outcomes. This underlines that the multi-faceted challenge OAC therapy can be successfully tackled by an eHealth-based, specialized CS.

In general, it is challenging to compare the present results with findings from previous studies due to variations in study settings (e.g. differences in social aspects and healthcare systems). The thrombEVAL eHealth based coagulation service as such is the first of its kind in Germany. Also, it must be recognized that not all anticoagulation management systems are the same (e.g. difference in qualifications of staff, support software)^21^. However, the overall cost saving of 2368 EUR per py in the CS cohort in the present analysis is similar to previously stated amounts. Aziz et al.^18^ reported a yearly net saving of 2311 EUR per py (adjusted for inflation and converted into EUR with currency rate of 2018/10/23), Chiquette et al.^14^ of 1897 EUR per py, and Rudd et al.^15^ of 998 EUR per py. Each of the mentioned studies has clear limitations; nevertheless, the hypothesis of a coagulation service being cost-saving is substantiated. In most investigations^14,15,22^, but not all^17^, there is a lack of detailed management costs of the CS as well as reliable actual financial data from medical claims. However, these shortcomings were addressed in the present study and, in addition, reasons for hospital admissions and the associated costs were evaluated for both cohorts. Of note, the formally structured coagulation service has been shown to improve the clinical outcome of patients when compared to usual medical care (e.g. lower rate of hospitalisations, major and clinically relevant bleeding and all-cause mortality)^12^. It must be pointed out that investigations stating less auspicious results regarding the beneficial effect of CS on therapeutic outcome did not conduct cost-saving analyses, so potential publication bias cannot be ruled out with certainty^23–25^.

An improved control regarding quality of anticoagulation, assessed by the TTR, seems to be the key player in improved clinical outcome in the CS^12,26^. Generally, if anticoagulation therapy is managed by a CS, subjects are less likely to sustain bleeding or venous/arterial thromboembolic complications leading to less OAC related-hospitalisation^13,14^, which can also be observed independent of the TTR^12^. Of note, in literature^15,17,18^ but also in the present study even more pronounced differences in non-OAC related hospitalisations between cohorts were depicted. This supports the hypothesis that a specialised individually tailored healthcare model (i.e. organized standardized-driven care, detailed standardized assessment of clinical status at each visit, and individualized termination of control visits depending on medical necessity) allows evolving health problems to be identified early and possibly addressed at the CS visit. This is underlined by the most frequent reasons for hospital admission. For example, subjects in CS were significantly less often discharged with a diagnosis of unspecified atherosclerosis which is not related to the therapy with oral anticoagulants. Overall, a combination of improved TTR, regular contact with medical staff to early detect clinical symptoms, good patient education and high availability of specialists for potential questions seem certainly as the most promising explanation for a reduction of hospitalisations which in turn leads to lower costs in CS compared to RMC. It can be speculated that preventive measures encouraged by CS also reduce costs per admission for certain diagnoses as seen in the current analysis. However, this fact has not been investigated so far and remains to be evaluated in more detail.

Since DOAC are currently the most frequently prescribed oral anticoagulant agent^27^, it is of importance to set the present results into context. Recent studies estimated the differences in medical costs for clinical outcomes associated with the use of DOAC versus VKA when at different TTR levels. Despite higher drug costs, DOAC reduced overall medical costs compared to warfarin, unless the TTR for VKA is above 60%^28^ or 65%^29^. Large reductions in medical costs were mainly driven by less major bleedings with the DOAC^30^. A TTR in VKA-treated patients of 75%, as in the current CS-cohort, emphasizes the assumption that VKA treatment in CS is superior to DOAC with regard to cost-saving. It has to be noted that a TTR of ≥ 70% is often difficult to maintain in daily regular medical care, as shown in registry-based studies^31^ as well as randomized clinical trials^32^. In this regard, considerations could be given to the possibility of managing patients receiving DOAC in a specialized service to also saving from lower frequency of adverse events associated to this alternative form of health care provision.

### Limitations

Some limitations of this work need to be acknowledged. First, the use of a non-randomized study design might have introduced selection bias. Against this background, propensity score weighted analysis accounting for clinical differences between cohorts was performed which proved the robustness of results. Generally, randomization to healthcare models seems problematic in real-life investigations, particularly because of the limited ability to fully investigate a real-life scenario, but also because of patients´ beliefs and logistic and financial reasons. Second, the basis for the assessment of management costs differed between the two groups. In RMC, management costs were based on the reimbursement of costs by the national health insurance only (there is a higher reimbursement for individuals with private health insurance), whereas costs in CS were equal for all patients and were based on invoices of the service provider and real personnel costs. Since the percentage of individuals with private health insurance is low (approx. 10% in Germany)^33^), the actual difference in costs between the two systems is expected to be slightly underestimated. Third, costs for outpatient visits and procedures as well as rehabilitation were not considered. Given the higher event rate in RMC vs. CS, an even greater cost difference is likely if ambulatory costs would be included. Fourth, the follow-up period of 12-months was relatively short but nevertheless greater than in previous investigations^17^. However, it can be seen that hospital admission rates decreased with time and therefore one might suspect an even greater net saving for the CS compared to RMC with longer observational time. Fifth, the majority of subjects (> 98%) received phenprocoumon for anticoagulation therapy, which limits extrapolation to other oral anticoagulants. It is to be assumed that one can see at least similar savings since therapy with warfarin is associated with greater INR variability. Last, the CS was operated by a University Medical Center with specialized hemostaseological expertise compared to routine medical care provided by ambulatory working general practitioners and specialized physicians, which might have enhanced the difference between both care models.

## Conclusion

The present study illustrates that the improvement of clinical outcome of anticoagulated patients by a specialized, eHealth-based anticoagulation management system is cost-effective. Since cost saving was achieved by a reduction of both OAC-specific and non-specific costs, this specialized health care model may also be beneficial for patients receiving other anticoagulant agents. Generally, these findings could also stimulate the evaluation of actions to improve quality of care in further medical fields to avoid higher secondary costs for the health care system.

## Methods

### Study design

The thrombEVAL study (NCT01809015) is a prospective multi-centre cohort study comprising two prospective cohorts: a cohort of patients receiving oral anticoagulation therapy in regular medical care (RMC) and a cohort of anticoagulated patients whose anticoagulation therapy is managed by an eHealth-based, specialized coagulation service (CS). Rationale and design of the study have been previously published^34^. The aim of the study was to investigate the quality of OAC with VKA in current health care and to evaluate the potential for improvements, and set it into context to a cost-saving analysis. Sample size calculations were conducted to determine the optimal sample size and thereof assure an adequate power to detect statistical significant differences between groups. All procedures were performed according to the principles of the Declaration of Helsinki, good clinical practice and good epidemiological practice. Approval of the local ethics committee (federal medical association Rhineland-Palatinate, reference no. 837.407.10.7415/7416) was obtained for all sites. All participants provided informed written consent prior to study enrolment.

### Study setting and study population

The study base of the thrombEVAL study is constituted by a Western European, predominantly white population. Patients were recruited from January 2011 to March 2013. Patients were eligible if they had the indication for OAC therapy for at least a further 3 months. The allocation to the cohorts was not controlled or influenced by investigators of the study and was subject to the autonomous decision of the patients (or their legal representative, if appropriate). Study enrolment for the multicentre cohort of RMC (N = 2011 subjects) was carried out at hospitals and oral anticoagulation therapy was provided by general practitioners and medical specialists (e.g. internal medicine). Due to the observational nature of the study, management of OAC therapy as well as any other medical decisions were not influenced by the investigators of the study and were left at the physicians´ discretion.

In the CS cohort (N = 760 subjects), management of OAC therapy was performed by a specialized service that provided expert care by specifically-trained staff (i.e. nurses, medical doctors and senior physicians). Visits for anticoagulation control were conducted in a highly standardized fashion with individualized time intervals according to medical necessity. All treatment information (i.e. standardized assessment of clinical status and level of international normalized ratio [INR] at each visit) was documented decentralized in a web-based, digital case report form (Portavita B.V., Amsterdam, the Netherlands). This facilitated the coverage of both urban and rural areas and enabled near-patient health care provision with standardized clinical assessment and digital documentation. The electronic file was accessible for staff, patients and all physicians in charge (dependent on the patient´s consent) via a secured internet connection. In the CS cohort, anticoagulant dose-adjustment was based on the use of electronic patient file data and integrated computer-assisted dosing algorithms. Automated scheduling of OAC control visits was established to prevent loss to follow-up and improve patient adherence. The RMC group is a purely observational prospective study, so no interventions on therapy adjustment and therapy monitoring were conducted.

In the RMC cohort, clinically relevant events were documented in electronic case report forms via annual computer-assisted telephone interviews, whereas in the CS outcome-relevant events were recorded in the electronic patient file within the framework of anticoagulation control visits. Information on study endpoints was validated by medical records and adjudicated by an independent review panel. In addition, electronic database systems of hospital records were screened for unreported events to reduce a potential recall bias. All data were checked for completeness, plausibility and validity according to pre-specified criteria which are defined in a data management plan Median follow-up times for RMC and the CS were 12.0 (IQR, 11.9/12.0) and 14.0 (IQR, 6.6/19.2) months, respectively.

### Data collection and analysis

#### Costs for oral anticoagulation management

The RMC management costs are based on cost of INR measurements that are determined via the health care system (i.e. statutory scale of fees for physicians^20^). Of note, costs for INR testing in Germany vary with the insurance status of the patient, meaning statutory health insurance or private health insurance from a German or international insurance company. The current analysis was based on costs in the statutory health care system, where the majority of patients are part of (approx. 90%)^33^. Estimated INR testing frequency of 24.0 times per year for CS testing was assumed based on published data from this cohort^26^.

Operational costs of the CS cohort consisted of compensation for staff, preparation and dispatch of dosage recommendations and licensing fees for the electronic patient file (Portavita B.V) during the study period. The personnel cost component per blood testing consisted of 12 min nursing time per contact with the patient (for blood withdrawal, collection and entry of treatment-relevant information in the electronic file) at 31.55 € per hour and 5 min medical doctor time per contact with the patient (for VKA dosing) at 54.92 € per hour. The INR testing frequency of 21.5 times per year for usual care was derived from previously published data on the thrombEVAL study^26^. In the current analysis medication costs for VKA were not included since they are assumed to be equal in both cohorts.

#### Costs for hospitalisation

Based on information on the clinical outcome of participants in both cohorts, the associated hospitalization costs were evaluated. Costs estimates were based on available physician’s letters which provide a blanket coverage of all hospitalisation costs. Diagnoses, operations and medical procedures were coded according to the German modification of the ICD-10 (ICD-10GM) and the operation and procedure key codes (OPS). OPS are provided by InEK (Institute for the Hospital Remuneration System) on behalf of the self-governing partners of the German health care and are used in inpatient care. Both are essential components in the diagnosis-related group (DRG) codes on which the calculation of total costs per hospital stay is based on. The DRG system is a medico-economic patient classification system that reproduces the services provided by hospitals via a fee-for-service payment system^35^. All discharged hospital patients are assigned to a DRG code using on an algorithm which is based on inpatient hospital discharge dataset, containing: major and other diagnosis, medical procedures, patient characteristics (age, gender), length of hospital stay, reason for hospital dis-charge and type of admission (e.g. emergency, referral from GP or transfer from other hospital). For each of the DRG codes a specific economic case value has been prescribed, and this case value, multiplied with a base rate which is up to now specific for each hospital produces the reimbursement for a specific patient.

### Statistical analysis

Discrete variables were described by absolute and relative frequencies, and continuous variables by means with standard deviation or median value with 25th/75th percentiles (interquartile range, IQR), where appropriate. No imputation was performed to address drop-out data. The time in therapeutic range (TTR) was calculated according to the Rosendaal Method^36^. Hospital admissions within the first 12 months of the study period were considered for analysis. In order to avoid bias, the first 2 months of follow-up were blanked for the cost-saving analysis since study enrolment for the RMC cohort was performed during a hospitalization of the patient. In order to reduce the possible bias (higher probability of complications and / or re-hospitalizations shortly after inclusion in the study) as a result, a blanking period was introduced. It was distinguished between hospital costs resulting from complications of anticoagulation (i.e. clinically-relevant bleeding or thromboembolic events) and hospital costs which were not related to anticoagulation treatment (i.e. any other reason for admission or death). To obtain total hospitalisation costs per patient year, recorded costs for 10 months were extrapolated to 12 months and divided by the total number of participants. Due to extreme skewness of the data, bootstrapping with 2000 replications was used to compute confidence intervals. Inverse probability of treatment weighting using a propensity score was performed under consideration of the cardiovascular profile (i.e. presence of traditional cardiovascular risk factors and history of cardiovascular disease). Cost-saving calculations were performed by using the differences in total costs between RMC and CS. All statistical analyses were conducted using the software program R, v.3.1.1 (http://www.r-project.org).

## Abbreviations

CS: Coagulation service
DOAC: Direct acting anticoagulants
DRG: Diagnosis-related group
INR: International normalised ratio
IQR: Interquartile range
OAC: Oral anticoagulation
OPS: Operation and procedure key codes
py: Patient-years
RMC: Regular medical care
TTR: Time in therapeutic range
VKA: Vitamin K antagonists
